# Exploring Clinical Similarities and Distinctions Between Gastroparesis and Functional Dyspepsia: A Propensity‐Matched Cohort Study

**DOI:** 10.1111/nmo.70251

**Published:** 2026-01-23

**Authors:** Sameer Rao, Vraj Shah, Rohan Karkra, Manas Gunani, Ritik Mahaveer Goyal, Ahmed Al‐Khazraji, Kaveh Hajifathalian, Amanda A. Rupert

**Affiliations:** ^1^ Department of Medicine Rutgers New Jersey Medical School New Jersey USA; ^2^ Department of Medicine Allegheny Health Network Pennsylvania USA; ^3^ Division of Gastroenterology and Hepatology Rutgers New Jersey Medical School New Jersey USA

**Keywords:** disorders of gut brain interaction, functional dyspepsia, gastric emptying, gastroparesis, prokinetics

## Abstract

**Background and Aims:**

Gastroparesis and functional dyspepsia often share overlapping upper gastrointestinal symptoms but may differ in symptom patterns, treatment, and health care utilization. We assessed their clinical similarities and distinctions in the largest national cohort study to date.

**Methods:**

We performed a retrospective cohort study using the TriNetX database, identifying patients through administrative codes. Individuals with structural gastric or small bowel abnormalities or prior gastric surgery were excluded. Gastroparesis required a diagnosis within three months of a gastric emptying study, at least one typical symptom, and an upper endoscopy within 12 months prior. Functional dyspepsia required a diagnosis with prior endoscopy (1–12 months) and symptoms 6–12 months before diagnosis. Primary outcomes were medication use and healthcare utilization; secondary outcomes included symptom burden and coexisting disorders of gut–brain interaction. Propensity score matching (1:1) adjusted for demographics and comorbidities. Relative risks with 95% confidence intervals were calculated.

**Results:**

We identified 2488 patients with gastroparesis and 3676 with functional dyspepsia; after matching, 1914 per group remained. Gastroparesis showed greater use of prokinetics (52.7% vs. 19.6%; *p* < 0.0001) and antiemetics, and higher rates of endoscopy (41.4% vs. 21.2%), emergency visits (45.3% vs. 40.2%), and hospitalization (28.2% vs. 21.6%) (all *p* < 0.01). Nausea, vomiting, and distension were more frequent in gastroparesis, while epigastric pain predominated in functional dyspepsia (*p* < 0.0001).

**Conclusion:**

Gastroparesis and functional dyspepsia show distinct symptom distributions and treatment patterns, with higher health care utilization in gastroparesis, supporting mechanism‐based individualized management.

## Key Points

Despite overlapping upper gastrointestinal symptoms, gastroparesis and functional dyspepsia demonstrate distinct **population‐level** symptom patterns, with nausea, vomiting, and bloating predominating in gastroparesis and epigastric pain in functional dyspepsia. Gastroparesis is also associated with greater use of prokinetics and antiemetics and higher healthcare utilization, supporting diagnostic distinction to enable targeted management.

## Graphical abstract

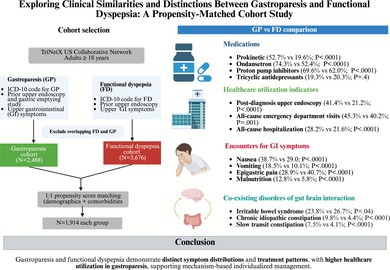

## 1 Introduction

Gastroparesis (GP) and functional dyspepsia (FD) are among the most prevalent upper gastrointestinal sensorimotor disorders in adults and represent a significant burden to both patients and health care systems worldwide [1, 2]. Gastroparesis is characterized by delayed gastric emptying in the absence of mechanical obstruction, with symptoms including nausea, vomiting, early satiety, postprandial fullness, bloating, and epigastric pain [3]. In contrast, functional dyspepsia is defined by the Rome IV criteria as a disorder of gut–brain interaction diagnosed in the absence of any identifiable organic etiology. It is characterized by chronic or recurrent upper abdominal pain or discomfort, postprandial fullness, and early satiety [4]. The standardized prevalence of gastroparesis ranges from 13.8 to 267.7 per 100,000 adults [5, 6], while functional dyspepsia is estimated to affect approximately 7200 per 100,000 of adults globally based on the Rome IV criteria, with rates reaching as high as 37.9% in low‐ and middle‐income settings [7, 8] Both disorders disproportionately affect women and are associated with substantial reductions in quality of life, increased health care utilization, and a high prevalence of psychological comorbidities [9, 10].

The clinical presentations of gastroparesis and functional dyspepsia overlap considerably, with both conditions sharing several key symptoms, including early satiety, bloating, postprandial fullness, and upper abdominal pain, which often makes them difficult to distinguish clinically [11]. Despite these similarities, vomiting and weight loss are more characteristic of gastroparesis, particularly in advanced stages, whereas epigastric discomfort and pain are more prominent in functional dyspepsia, especially in the epigastric pain syndrome subtype [12, 13]. The pathophysiology of both disorders is multifactorial and remains incompletely understood, involving abnormalities in gastric motility (such as delayed emptying and impaired accommodation), visceral hypersensitivity, mucosal inflammation, immune dysregulation, and disturbances of the gut–brain axis [14, 15]. Emerging evidence suggests that gastroparesis and functional dyspepsia may exist along a continuum of gastric neuromuscular dysfunction rather than as entirely distinct entities [16].

Despite growing awareness and improved diagnostic tools, the close clinical and pathophysiological similarities between functional dyspepsia and gastroparesis continue to pose major challenges for diagnosis, management, and prognosis. Symptom‐based criteria and gastric emptying studies are limited by a poor correlation between symptom severity and motor dysfunction, as well as by inconsistent validity on repeat testing [3, 16]. This overlap frequently leads to misclassification, underdiagnosis, and variability in management, particularly in primary care settings where awareness of, and access to, specialized diagnostic testing may be limited [17]. These challenges have important clinical implications, as gastroparesis is associated with greater risks of nutritional compromise, hospitalization, and mortality, whereas functional dyspepsia generally carries a more favorable prognosis but remains a common cause of chronic gastrointestinal symptoms, patient frustration, and increased health care utilization [6, 18].

To evaluate these challenges, we conducted the largest head‐to‐head comparative study of key clinical domains between gastroparesis and functional dyspepsia, leveraging data from the U.S. Collaborative Health Network within the TriNetX platform, which broadly reflects the demographic and clinical diversity of the U.S. population. We hypothesized that although the two disorders share substantial symptom overlap, distinct differences in symptom patterns, health care utilization, and patient‐reported outcomes would emerge after accounting for baseline covariates. Clarifying these similarities and differences has direct implications for diagnosis, management, and research. Greater diagnostic specificity can improve early recognition of at‐risk patients, guide targeted therapies, and optimize health care resources. Furthermore, distinguishing shared and unique pathophysiological mechanisms may inform development of mechanism‐based treatments and integrated care strategies.

## 2 Methods

### 2.1 Database

TriNetX is a federated research network that provides access to deidentified electronic health records from more than 150 health care organizations across the United States (US) and globally. This study utilized data exclusively from the United States Collaborative Network, which comprises 67 participating sites, including academic medical centers, specialty physician groups, and integrated health care systems across the country. The database captures real‐time clinical information such as patient demographics, clinical diagnoses, medical procedures, prescription medications, laboratory test results, and mortality. All data are standardized using established clinical coding systems, including the International Classification of Diseases (ICD), Current Procedural Terminology (CPT), Logical Observation Identifiers Names and Codes (LOINC), and RxNorm. As a federated system, TriNetX protects patient privacy by conducting analyses within the secure environments of participating institutions and returning only aggregated, nonidentifiable results. This makes it a widely used platform for large‐scale observational research within the United States.

### 2.2 Study Design

We conducted a retrospective cohort study of adults in the United States using the TriNetX database, identifying clinical attributes through ICD‐10, CPT, LOINC, and RxNorm codes. Institutional review board (IRB) approval was not required, as the study utilized deidentified data. We queried patients with records in the TriNetX database from January 1, 2015, to December 31, 2024.

Patients with structural abnormalities of the stomach or small intestine, including gastritis, duodenitis, gastric or duodenal ulcers, gastric outlet obstruction, stomach cancer, intestinal obstruction, congenital malformations such as pyloric stenosis or intestinal atresia, intussusception, volvulus, and adhesions were excluded prior to cohort formation. Additionally, patients with a history of gastric surgery, including gastrectomy, pyloromyotomy, gastrojejunostomy, sleeve gastrectomy, or gastric bypass were excluded.

**FIGURE 1:**
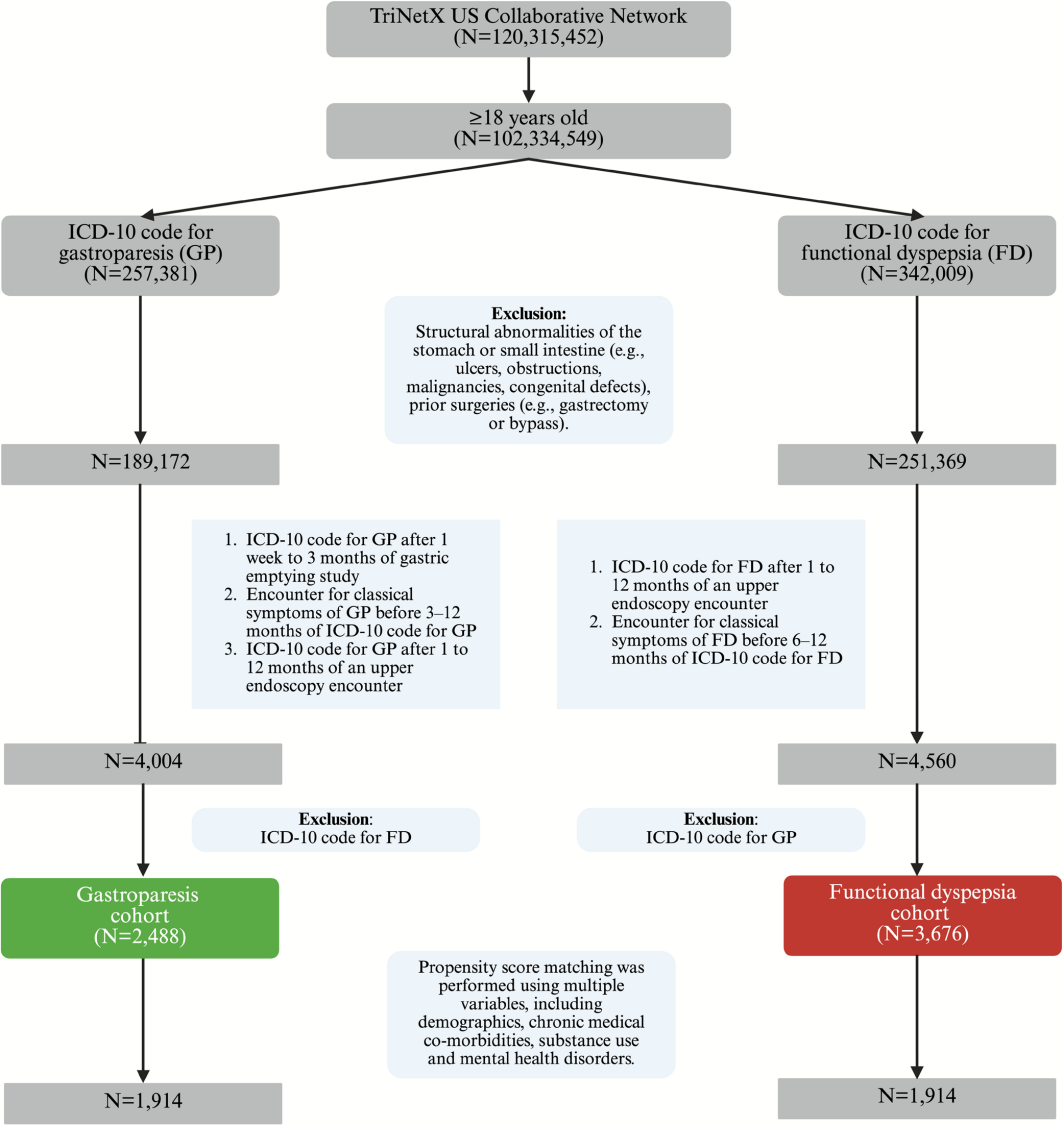
Flowchart depicting cohort selection for gastroparesis and functional dyspepsia using the TriNetX US Collaborative Network. FD—functional dyspepsia, GP—gastroparesis, and ICD‐10—international classification of diseases, 10th revision.

The gastroparesis cohort was defined by a diagnosis of gastroparesis (ICD‐10 code K31.84) recorded within 1 week to 3 months after a gastric emptying study, as quantitative gastric emptying results were not available in the database, and by the presence of at least one typical symptom—such as nausea, vomiting, epigastric pain, early satiety, postprandial fullness, or abdominal bloating occurring three to 12 months prior to diagnosis. Additionally, patients should have had an upper endoscopy within one to 12 months prior to diagnosis. The functional dyspepsia cohort was defined by a diagnosis of functional dyspepsia (ICD‐10 code K30) with a preceding upper endoscopy performed one to 12 months earlier and the presence of typical symptoms—such as epigastric pain, early satiety, or postprandial fullness—occurring six to 12 months prior. Abdominal bloating was used as a proxy for postprandial fullness due to the lack of a specific ICD 10 code. Patients with overlapping diagnoses of gastroparesis and functional dyspepsia were excluded (Figure 1).

To reduce potential bias from reverse causation and diagnostic clustering, we excluded outcomes that occurred within the first 30 days following the index diagnosis. The primary outcomes were medication use and health care utilization. Secondary outcomes included symptom burden, coexisting disorders of gut–brain interaction, and all‐cause mortality. The ICD‐10, CPT, and RxNorm codes used to define all variables are provided in the Table S1.

### 2.3 Statistical Analysis

All analyses were carried out using the built‐in tools available on the TriNetX platform, which supports real‐time cohort construction and comparison across participating health care institutions. To reduce baseline differences between groups, we performed one‐to‐one propensity score matching using a nearest‐neighbor method with a caliper set at 0.1 times the pooled standard deviation. Variables included in the propensity score model were selected based on clinical relevance and included age, sex, race and ethnicity, body mass index, gastroesophageal reflux disease, celiac disease, inflammatory bowel disease (Crohn's disease and ulcerative colitis), liver disease (fibrosis or cirrhosis and fatty liver disease), substance use disorders (alcohol, cannabis, opioid, and nicotine dependence), mental health disorders (anxiety, depression, posttraumatic stress disorder, bipolar disorder, adjustment disorder, and schizophrenia), and systemic comorbidities including dyslipidemia, hypertension, overweight or obesity, hypothyroidism, diabetes mellitus (type 1 and type 2), chronic kidney disease, ischemic heart disease, prior myocardial infarction, cerebrovascular disease, heart failure, asthma, chronic obstructive pulmonary disease, sleep apnea, and dependence on renal dialysis or mechanical ventilation. Following matching, categorical variables were compared using the chi‐squared test, while continuous variables were assessed with independent *t*‐tests. Results for binary outcomes were expressed as relative risks (RRs) with 95% confidence intervals (CIs). A *p*‐value < 0.05 was used to define statistical significance.

## 3 Results

Our study had 4004 patients with gastroparesis (mean age 51.9 ± 17.6 years; 78.7% female) and 4560 patients with functional dyspepsia (mean age 50.6 ± 20.0 years; 73.8% female) who met the inclusion criteria. In the gastroparesis cohort, patients with a coexisting ICD‐10 diagnosis of functional dyspepsia were excluded (*n* = 1516; 37.9%), resulting in a final cohort of 2488 patients (mean age 47.2 ± 17.5 years; 75.6% female). Similarly, in the functional dyspepsia cohort, patients with a coexisting ICD‐10 diagnosis of gastroparesis were excluded (*n* = 884; 19.4%), yielding a final cohort of 3676 patients (mean age 45.1 ± 20.3 years; 69.6% female). After 1:1 propensity score matching, 1914 patients per group were identified. The median duration of follow‐up in the gastroparesis cohort was 39.5 months with interquartile range of 50.1 months, while the median duration of follow‐up in the functional dyspepsia cohort was 40.7 months with interquartile range of 50.1 months.

Baseline characteristics of the gastroparesis and functional dyspepsia cohorts before and after propensity score matching are summarized in Table 1. Prior to matching, significant differences were observed across demographic distributions, with gastroparesis patients more frequently identifying as Black or African American, while functional dyspepsia patients had higher proportions of Hispanic or Latino and Asian individuals. Multiple comorbidities were more prevalent in the gastroparesis group, including hypertension, type 1 diabetes, type 2 diabetes, chronic kidney disease, heart failure, ischemic heart disease, obesity, hypothyroidism, chronic obstructive pulmonary disease, and sleep apnea. Psychiatric conditions such as depression, anxiety, bipolar disorder, and schizophrenia were also more frequently observed. Substance use disorders, including nicotine dependence, cannabis use, and opioid use, were significantly more common among gastroparesis patients. After propensity score matching, baseline characteristics were well balanced across demographic, clinical, psychiatric, and substance use variables, although a small residual difference in body mass index remained (28.8 vs. 28.2 kg/m^2^; *p* < 0.0001).

**TABLE 1:** Baseline characteristics of the gastroparesis and functional dyspepsia cohorts before and after propensity score matching.

	Characteristic	GP N (%)	FD N (%)	*p*
For each attribute, upper and lower row represents pre‐ and post‐propensity match data respectively				
Demographics	Age at index	Mean 47.2 ± SD 17.5 (*N* = 2488)	Mean 45.1 ± SD 20.3 (*N* = 3676)	**< 0.0001**
		Mean 46.3 ± SD 18 (*N* = 1914)	Mean 47.1 ± SD 19.8 (*N* = 1914)	0.1848
	Female	1880 (75.56)	2557 (69.39)	**< 0.0001**
		1436 (75.03)	1424 (74.40)	0.6554
	White	1734 (69.69)	2514 (68.39)	0.2774
		1369 (71.53)	1355 (70.79)	0.6174
	Black or African American	383 (15.39)	325 (8.84)	**< 0.0001**
		228 (11.91)	236 (12.33)	0.6920
	Hispanic or latino	164 (6.59)	366 (9.96)	**< 0.0001**
		128 (6.69)	138 (7.21)	0.5250
	Asian	38 (1.53)	198 (5.39)	**< 0.0001**
		38 (1.98)	40 (2.09)	0.8190
	Other race	104 (4.18)	164 (4.46)	0.5952
		84 (4.39)	85 (4.44)	0.9373
Body mass index		Mean 29.1 ± SD 8.48 (*N* = 1991)	Mean 27.1 ± SD 7.14 (*N* = 3054)	**< 0.0001**
		Mean 28.8 ± SD 8.28 (*N* = 1547)	Mean 28.2 ± SD 7.48 (*N* = 1524)	**< 0.0001**
Gastrointestinal diseases	GERD	1744 (70.10)	2473 (67.27)	**0.0194**
		1327 (69.33)	1352 (70.64)	0.3780
	Celiac disease	52 (2.09)	64 (1.74)	0.3225
		41 (2.14)	44 (2.30)	0.7421
	Crohn's disease	54 (2.17)	56 (1.52)	0.0598
		36 (1.88)	39 (2.04)	0.7264
	Ulcerative colitis	45 (1.81)	68 (1.85)	0.9059
		35 (1.88)	32 (2.04)	0.7264
	Fibrosis or cirrhosis of liver	88 (3.54)	103 (2.80)	0.1023
		58 (3.03)	67 (3.50)	0.4131
	Fatty liver disease	334 (13.42)	399 (10.85)	**0.0022**
		224 (11.70)	236 (12.33)	0.5508
Substance use disorders	Alcohol use disorder	102 (4.1)	133 (3.62)	0.3327
		67 (3.50)	73 (3.81)	0.6054
	Cannabis use disorder	147 (5.91)	92 (2.50)	**< 0.0001**
		83 (4.34)	81 (4.23)	0.8732
	Opioid use disorder	124 (4.98)	77 (2.09)	**< 0.0001**
		66 (3.45)	68 (3.55)	0.8604
	Nicotine dependance	441 (17.72)	407 (11.07)	**< 0.0001**
		278 (14.52)	280 (14.63)	0.9270
Mental health disorders	Anxiety	1152 (46.30)	1483 (40.34)	**< 0.0001**
		834 (43.57)	855 (44.67)	0.4942
	Depression	997 (40.07)	1026 (27.91)	**< 0.0001**
		665 (34.74)	677 (35.37)	0.6844
	PTSD	205 (8.24)	289 (7.86)	0.5920
		127 (6.63)	130 (6.79)	0.8464
	Bipolar	195 (7.84)	124 (3.37)	**< 0.0001**
		101 (5.28)	110 (5.75)	0.5239
	Adjustment disorder	205 (8.24)	289 (7.86)	0.5920
		146 (7.63)	151 (7.89)	0.7626
	Schizophrenia	23 (0.92)	19 (0.49)	**0.0394**
		16 (0.84)	14 (0.73)	0.7139
Systemic conditions	Lipid disorders	1146 (46.06)	1304 (35.47)	**< 0.0001**
		752 (39.29)	749 (39.13)	0.9209
	Hypertension	1269 (51.00)	1238 (33.68)	**< 0.0001**
		797 (41.64)	808 (42.21)	0.7186
	Overweight or obesity	876 (35.21)	879 (23.91)	**< 0.0001**
		595 (31.09)	601 (31.40)	0.8343
	Hypothyroidism	418 (16.80)	483 (13.14)	**< 0.0001**
		284 (14.84)	301 (15.73)	0.4451
	Type 1 DM	387 (15.56)	98 (2.67)	**< 0.0001**
		114 (5.96)	98 (5.12)	0.2582
	Type 2 DM	990 (39.79)	547 (14.88)	**< 0.0001**
		481 (25.13)	485 (25.34)	0.8817
	Chronic kidney disease	449 (18.05)	222 (6.04)	**< 0.0001**
		161 (8.41)	158 (8.25)	0.8607
	Angina pectoris	150 (6.03)	151 (4.11)	**0.0006**
		89 (4.65)	96 (5.02)	0.5978
	Chronic ischemic heart disease	418 (16.80)	382 (10.39)	**< 0.0001**
		252 (13.27)	244 (12.75)	0.6309
	Acute MI	112 (4.50)	96 (2.61)	**< 0.0001**
		62 (3.24)	63 (3.29)	0.9275
	Cerebral infarction	114 (4.58)	92 (2.50)	**< 0.0001**
		71 (3.71)	73 (3.81)	0.6054
	Heart failure	280 (11.25)	191 (5.20)	**< 0.0001**
		153 (7.99)	142 (7.42)	0.5050
	Asthma	649 (26.08)	742 (20.18)	**< 0.0001**
		461 (24.09)	472 (24.66)	0.6788
	Sleep apnea	548 (22.03)	561 (15.26)	**< 0.0001**
		363 (18.97)	388 (20.27)	0.3089
	COPD	256 (10.29)	232 (6.31)	**< 0.0001**
		161 (8.41)	158 (7.42)	0.5050
	Dependence on renal dialysis	78 (3.13)	19 (0.52)	**< 0.0001**
		17 (0.89)	18 (0.94)	0.8652
	Dependence on respirator	21 (0.84)	13 (0.35)	**0.0108**
		10 (0.52)	10 (0.52)	1

*Note:* *P*‐values in bold font represent statistically significant results.

Abbreviations: BMI, body mass index; COPD, chronic obstructive pulmonary disease; DM, diabetes mellitus; FD, functional dyspepsia; GERD, gastroesophageal reflux disease; GP, gastroparesis; MI, myocardial infarction; PTSD, post‐traumatic stress disorder.

### 3.1 Medication Use and Health Care Utilization

Unadjusted and propensity score–matched comparisons of medication use and health care utilization are shown in Table 2, with matched analyses serving as the primary results and described below.

**TABLE 2:** Comparison of medication use and health care utilization between gastroparesis and functional dyspepsia cohorts before and after 1:1 propensity matching.

Clinical outcomes after propensity matching	GP N (%)	FD N (%)	Relative risk^a^	Confidence interval	*p*
For each attribute, upper and lower row represents pre‐ and post‐propensity match data respectively					
N of patients	2488	3676			
	1914	1914			
Medications					
Metoclopramide	1121 (45.07)	441 (12.00)	3.76	(3.40,4.15)	**< 0.0001**
	786 (41.07)	251 (13.11)	3.13	(2.76,3.56)	**< 0.0001**
Erythromycin	361 (14.51)	209 (5.68)	2.55	(2.16,3.01)	**< 0.0001**
	276 (14.42)	108 (5.64)	2.56	(2.06,3.16)	**< 0.0001**
Prucalopride	247 (9.93)	46 (1.25)	8.01	(5.82,11.03)	**< 0.0001**
	213 (11.13)	30 (1.57)	7.10	(4.87,10.35)	**< 0.0001**
Prokinetic^b^	1362 (54.73)	649 (17.65)	3.10	(2.86,3.36)	**< 0.0001**
	1024 (52.65)	380 (19.64)	2.69	(2.44,2.98)	**< 0.0001**
Ondansetron	1922 (77.24)	1837 (49.97)	1.55	(1.49,1.61)	**< 0.0001**
	1423 (74.35)	1003 (52.40)	1.42	(1.35,1.49)	**< 0.0001**
Prochlorperazine	763 (30.65)	510 (13.87)	2.21	(2.00,2.45)	**< 0.0001**
	475 (24.82)	270 (14.11)	1.76	(1.54,2.01)	**< 0.0001**
Promethazine	883 (35.50)	503 (13.68)	2.59	(2.35,2.86)	**< 0.0001**
	624 (32.60)	268 (14.00)	2.33	(2.05,2.65)	**< 0.0001**
Diphenhydramine	1052 (42.28)	889 (24.18)	1.75	(1.62,1.88)	**< 0.0001**
	718 (37.51)	454 (23.72)	1.58	(1.43,1.75)	**< 0.0001**
Dronabinol	90 (3.62)	23 (0.63)	5.68	(3.58,9.03)	**< 0.0001**
	61 (3.19)	14 (0.73)	4.36	(2.45,7.76)	**< 0.0001**
Aprepitant	106 (4.26)	72 (1.96)	2.17	(1.60,2.92)	**< 0.0001**
	92 (4.81)	38 (1.98)	2.42	(1.69,3.51)	**< 0.0001**
Histamine‐2 receptor antagonists	1082 (43.49)	1146 (31.18)	1.39	(1.30,1.49)	**< 0.0001**
	782 (40.86)	605 (31.61)	1.29	(1.19,1.41)	**0.0012**
Proton pump inhibitors	1793 (72.07)	2213 (60.20)	1.20	(1.15,1.24)	**< 0.0001**
	1332 (69.59)	1186 (61.96)	1.12	(1.07,1.18)	**< 0.0001**
Tricyclic antidepressants	488 (19.61)	812 (22.09)	0.89	(0.80,0.98)	**0.02**
	369 (19.28)	388 (20.27)	0.95	(0.84,1.08)	0.4407
SNRI	205 (8.24)	219 (5.96)	1.38	(1.15,1.66)	**0.0006**
	144 (7.52)	119 (6.22)	1.21	(0.96,1.53)	0.1102
SSRI	952 (38.27)	1027 (27.94)	1.37	(1.27,1.47)	**< 0.0001**
	681 (35.58)	571 (29.83)	1.19	(1.09,1.31)	**0.0002**
Gabapentin	910 (36.58)	887 (24.13)	1.52	(1.40,1.64)	**< 0.0001**
	649 (33.91)	507 (26.49)	1.28	(1.16,1.41)	**< 0.0001**
Pregabalin	362 (14.55)	241 (6.56)	2.22	(1.90–2.60)	**< 0.0001**
	232 (12.12)	138 (7.21)	1.68	(1.37,2.05)	**< 0.0001**
Utilization metrics					
All‐cause CT scan of abdomen	105 (4.22)	91 (2.48)	1.70	(1.28,2.25)	**0.0002**
	63 (3.29)	55 (2.87)	1.14	(0.80,1.63)	0.4544
All‐cause post‐diagnosis EGD	1066 (42.85)	804 (21.87)	1.96	(1.81,2.12)	**< 0.0001**
	792 (41.38)	406 (21.21)	1.95	(1.76,2.16)	**< 0.0001**
All‐cause emergency department visits	1266 (50.88)	1373 (37.25)	1.36	(1.28,1.44)	**< 0.0001**
	867 (45.30)	770 (40.23)	1.13	(1.05,1.21)	**0.0015**
All‐cause hospitalization	830 (33.37)	704 (19.15)	1.74	(1.60,1.90)	**< 0.0001**
	539 (28.16)	413 (21.58)	1.30	(1.17,1.46)	**< 0.0001**

*Note:* *P*‐values in bold font represent statistically significant results.

Abbreviations: CT, computed tomography; EGD, esophagogastroduodenoscopy; FD, functional dyspepsia; GP, gastroparesis; SNRI, serotonin‐norepinephrine reuptake inhibitor; SSRI, selective serotonin reuptake inhibitor.

^a^ The relative risk is calculated as gastroparesis versus functional dyspepsia.

^b^ Prokinetic – metoclopramide and/or erythromycin and/or prucalopride.

Following propensity score matching, use of prokinetic and antiemetic medications remained significantly higher in the gastroparesis cohort compared with functional dyspepsia. Metoclopramide was prescribed in 41.1% of gastroparesis patients versus 13.1% in functional dyspepsia (RR, 3.13; *p* < 0.0001), while erythromycin use was 14.4% versus 5.6% (RR, 2.56; *p* < 0.0001). Prucalopride prescriptions were also higher among gastroparesis patients (11.1% vs. 1.6%; RR, 7.10; *p* < 0.0001). Overall prokinetic use (metoclopramide, erythromycin, or prucalopride) was markedly higher in gastroparesis (52.7% vs. 19.6%; RR, 2.69; *p* < 0.0001).

Antiemetics such as ondansetron (74.4% vs 52.4%; RR, 1.42; *p* < 0.0001), prochlorperazine (24.8% vs 14.1%; RR, 1.76; *p* < 0.0001), promethazine (32.6% vs 14.0%; RR 2.33; *p* < 0.0001), diphenhydramine (37.5% vs 23.7%; RR, 1.58; *p* < 0.0001), dronabinol (3.2% vs 0.7%; RR, 4.36; *p* < 0.0001), and aprepitant (4.8% vs 2.0%; RR, 2.42; *p* < 0.0001) were also more frequently used in gastroparesis. Histamine‐2 receptor antagonist use was higher in gastroparesis (40.9% vs 31.6%; RR, 1.29; *p* = 0.0012), as was proton pump inhibitor use (69.6% vs 62.0%; RR, 1.12; *p* < 0.0001). Use of tricyclic antidepressants was not significantly different (19.3% vs 20.3%; RR, 0.95; *p* = 0.4407).

Health care utilization demonstrated higher all‐cause hospitalization rates among gastroparesis patients (28.2% vs 21.6%; RR, 1.30; *p* < 0.0001). Rates of postdiagnosis upper endoscopy were also significantly higher (41.4% vs 21.2%; RR, 1.95; *p* < 0.0001), as were emergency department visits (45.3% vs 40.2%; RR, 1.13; *p* = 0.0015). No significant differences were noted in abdominal computed tomography utilization (3.3% vs 2.9%; RR, 1.14; *p* = 0.4544).

### 3.2 Symptom Burden and Co‐Existing DGBI

After matching, a greater proportion of patients with gastroparesis had encounters for upper gastrointestinal symptoms compared to those with functional dyspepsia, including nausea (38.7% vs. 29.1%; RR, 1.33; *p* < 0.0001), vomiting (18.5% vs. 10.1%; RR, 1.82; *p* < 0.0001), anorexia (6.6% vs. 4.2%; RR, 1.57; *p* = 0.0010), and abdominal distension (26.7% vs. 19.3%; RR, 1.38; *p* < 0.0001). Conversely, encounters for epigastric pain were significantly less common among gastroparesis patients (28.9% vs 40.7%; RR, 0.71; *p* < 0.0001).

Nutritional complications were more prevalent in gastroparesis, including malnutrition (12.9% vs 5.8%; RR, 2.22; *p* < 0.0001), severe protein‐calorie malnutrition (6.3% vs 2.6%; RR, 2.47; *p* < 0.0001), and abnormal weight loss (16.0% vs 12.2%; RR, 1.22; *p* = 0.0118).

Among co‐existing disorders of gut–brain interaction, irritable bowel syndrome was slightly less common in gastroparesis (23.8% vs 26.7%; RR, 0.89; *p* = 0.0408), while chronic idiopathic constipation (9.8% vs 4.4%; RR, 2.20; *p* < 0.0001), slow transit constipation (7.5% vs 4.1%; RR, 1.85; *p* < 0.0001), and functional diarrhea (1.9% vs 1.2%; RR, 1.68; *p* = 0.0491) were more frequently observed. All‐cause mortality did not differ significantly between cohorts (5.8% vs 5.1%; RR 1.13; *p* = 0.3551) (Table 3).

**TABLE 3:** Comparison of clinical symptom encounters and co‐existing disorders of gut–brain interactions between cohorts of gastroparesis and functional dyspepsia before and after 1:1 propensity matching.

Clinical outcomes after propensity matching	GP N (%)	FD N (%)	Relative risk^a^	Confidence interval	*p*
For each attribute, upper and lower row represents pre‐ and post‐propensity match data respectively					
N of patients	2488	3676			
	1914	1914			
Clinical attributes					
Nausea	985 (39.60)	1031 (28.04)	1.41	(1.31,1.52)	**< 0.0001**
	740 (38.66)	556 (29.05)	1.33	(1.22,1.46)	**< 0.0001**
Vomiting	566 (22.75)	360 (9.79)	2.32	(2.05,2.63)	**< 0.0001**
	353 (18.49)	193 (10.08)	1.82	(1.56,2.16)	**< 0.0001**
Anorexia	161 (6.47)	160 (4.35)	1.49	(1.20–1.85)	**0.0003**
	126 (6.58)	80 (4.18)	1.57	(1.20,2.07)	**0.0010**
Early satiety	127 (5.10)	164 (4.46)	1.14	(0.91,1.43)	**0.2668**
	102 (5.33)	92 (4.81)	1.11	(0.84,1.46)	0.4612
Abdominal distension	608 (24.45)	711 (19.35)	1.26	(1.14,1.39)	**< 0.0001**
	511 (26.70)	369 (19.28)	1.38	(1.23,1.56)	**< 0.0001**
Epigastric pain	728 (29.27)	1462 (39.78)	0.74	(0.68,0.79)	**< 0.0001**
	553 (28.89)	779 (40.70)	0.71	(0.65,0.78)	**< 0.0001**
Malnutrition	380 (15.28)	185 (5.03)	3.03	(2.55,3.59)	**< 0.0001**
	246 (12.85)	111 (5.80)	2.22	(1.79,2.75)	**< 0.0001**
Severe protein‐calorie malnutrition	182 (7.32)	89 (2.42)	3.03	(2.35,3.90)	**< 0.0001**
	121 (6.32)	49 (2.56)	2.47	(1.78,3.42)	**< 0.0001**
Abnormal weight loss	419 (16.83)	428 (11.64)	1.44	(1.27,1.64)	**< 0.0001**
	307 (16.04)	252 (12.17)	1.22	(1.04,1.42)	**0.0118**
Aspiration pneumonitis	120 (4.82)	51 (1.39)	3.47	(2.50–4.84)	**< 0.0001**
	74 (3.87)	30 (1.57)	2.47	(1.62,3.75)	**< 0.0001**
Coexisting DGBI					
Irritable bowel syndrome	582 (23.39)	1023 (27.82)	0.84	(0.77,0.92)	**0.0001**
	456 (23.82)	511 (26.70)	0.89	(0.80,0.99)	**0.0408**
Chronic idiopathic constipation	250 (10.05)	166 (4.51)	2.22	(1.83,2.69)	**< 0.0001**
	187 (9.77)	85 (4.44)	2.20	(1.72,2.82)	**< 0.0001**
Slow transit constipation	208 (8.36)	134 (3.65)	2.30	(1.86,2.86)	**< 0.0001**
	144 (7.52)	78 (4.07)	1.85	(1.41,2.41)	**< 0.0001**
Functional diarrhea	44 (1.77)	39 (1.06)	1.65	(1.07,2.55)	**0.0220**
	37 (1.93)	22 (1.15)	1.68	(0.99,2.84)	**0.0491**
All‐cause mortality	188 (7.56)	163 (4.44)	1.70	(1.39,2.10)	**< 0.0001**
	111 (5.80)	98 (5.12)	1.13	(0.87,1.47)	0.3551

*Note:* *P*‐values in bold font represent statistically significant results.

Abbreviations: DGBI, disorders of gut–brain interaction; FD, functional dyspepsia; GP, gastroparesis.

^a^ The relative risk is calculated as gastroparesis versus functional dyspepsia.

## 4 Discussion

There has been significant discussion and ongoing debate regarding the relationship between gastroparesis and functional dyspepsia, specifically whether they represent distinct clinical entities or a continuum of the same underlying disorder [11, 16, 19]. Our study contributes to the existing literature by providing the first large‐scale, national‐level analysis of gastroparesis and functional dyspepsia in the United States. The findings highlight significant differences in demographics, clinical outcomes, pharmacologic management, and health care utilization between these two entities.

To select our cohorts, we mirrored diagnostic criteria and standards as defined by current national guidelines and previously published studies. Specifically, the gastroparesis cohort was defined by a diagnosis of gastroparesis within three months of a gastric emptying study, the presence of at least one typical symptom, and an upper endoscopy performed within 12 months prior to diagnosis. This approach aligns with the American Gastroenterological Association (AGA) Guideline for Gastroparesis [5, 20]. The functional dyspepsia cohort was defined by a diagnosis with a preceding upper endoscopy within 12 months and the presence of typical symptoms, consistent with Rome IV criteria and prior studies [9, 21]. Since all included patients underwent a recent upper endoscopy, we were able to exclude those with structural abnormalities.

Patients with gastroparesis and functional dyspepsia demonstrated significant demographic differences, consistent with previous studies. Gastroparesis patients were older, more likely to be female, more likely to be Black, less likely to be Hispanic, and more likely to have multiple medical and psychiatric comorbidities [10, 11, 16]. These differences were adjusted for using propensity score matching.

Although there was considerable overlap in clinical symptoms between gastroparesis and functional dyspepsia, with both commonly presenting with nausea, vomiting, distension, and epigastric pain, we identified clear differences in the distribution of these symptoms as described in the existing literature [11, 22]. Patients with gastroparesis were more likely to present with nausea, vomiting, and distension, whereas those with functional dyspepsia were more likely to have epigastric pain. These findings emphasize that the symptom distribution differs significantly at the population level. Such differences not only aid in distinguishing the two disorders clinically but also translate into divergent therapeutic approaches.

For gastroparesis management, current guidelines recommend initial optimization of nutrition, favoring a small‐particle, low‐fat, low‐fiber diet followed by prokinetic agents such as metoclopramide or erythromycin when dietary modification alone is insufficient [1, 20, 23]. In accordance with these recommendations, our study found that patients with gastroparesis were approximately two to three times more likely than those with functional dyspepsia to receive a prokinetic agent. However, only about half of all patients with gastroparesis were prescribed one, indicating significant underutilization of guideline‐directed therapy. This underuse may partly reflect clinician concern about medication safety, as metoclopramide carries a boxed warning for tardive dyskinesia and erythromycin is associated with tachyphylaxis and potential QT prolongation [24, 25]. Additionally, prucalopride has been increasingly explored as an investigational prokinetic agent, particularly in idiopathic disease and in the presence of concomitant constipation [20, 26, 27]. Our study is the first to characterize real‐world prucalopride utilization in a large gastroparesis cohort, with approximately 11% of patients receiving prucalopride at some point during their care.

Nevertheless, given the high rates of hospitalization and emergency department use observed in this population [28], improving adherence to guideline‐based therapy and addressing barriers to appropriate pharmacologic management remain essential for optimizing patient outcomes. Recent data further delve into factors within gastroparesis that contribute to greater health care utilization, identifying diabetic etiology, delayed gastric emptying, younger age, Black race, lower income, and higher depression scores as key predictors of increased emergency department visits and hospitalizations [29]. Together, these findings highlight the complex interplay of clinical, psychological, and socioeconomic factors driving disease burden in gastroparesis and underscore the importance of a comprehensive, multidisciplinary management approach.

In contrast to gastroparesis, the management of functional dyspepsia primarily emphasizes *Helicobacter pylori* eradication (when present) and an empiric proton pump inhibitor trial as first‐line therapy, with neuromodulation as a second‐line approach, in accordance with the American College of Gastroenterology and Canadian Association of Gastroenterology guidelines [21]. Although prescriptions for tricyclic antidepressants and serotonin–norepinephrine reuptake inhibitors were comparable between gastroparesis and functional dyspepsia in our study, their use in gastroparesis is not guideline‐directed and may reflect empiric treatment for overlapping symptoms or associated psychiatric comorbidities rather than evidence‐based management [1, 10].

Differences in pharmacologic management between gastroparesis and functional dyspepsia are consistent with their divergent, though potentially overlapping, pathophysiologic mechanisms. Gastroparesis is believed to be primarily characterized by neuromuscular dysfunction, including injury to enteric neurons, loss of interstitial cells of Cajal, and impaired nitrergic signaling, resulting in delayed gastric emptying [30, 31, 32]. In contrast, functional dyspepsia is associated with visceral hypersensitivity, impaired duodenal barrier integrity, and low‐grade mucosal inflammation, with increased eosinophil and mast cell infiltration [33, 34]. Notably, delayed gastric emptying has been reported in some patients with functional dyspepsia, whereas normal emptying may occur in a subset with gastroparesis [16, 35]. These findings suggest overlapping sensorimotor dysfunction and support the concept that gastroparesis and functional dyspepsia may exist along a motility–sensory spectrum, underscoring the importance of tailoring therapy to the predominant pathophysiologic mechanism.

Our study also revealed substantial overlap of gastroparesis and functional dyspepsia with other disorders of gut–brain interaction. Prior studies from the NIH Gastroparesis Consortium and other cohorts have similarly reported high rates of overlapping DGBIs and psychological comorbidities in both disorders, supporting a shared pathophysiologic substrate involving altered motility, visceral sensitivity, and brain–gut axis dysregulation [36, 37, 38, 39]. However, our study demonstrates distinct population‐level patterns of overlap, with constipation‐predominant DGBIs clustering more strongly with gastroparesis and irritable bowel syndrome more frequently coexisting with functional dyspepsia. Recognizing such overlap is important, as coexisting DGBIs can compound symptom burden, complicate diagnosis, and influence therapeutic responsiveness.

The increasing recognition of both shared features and heterogeneity within gastroparesis and functional dyspepsia underscores the need to refine disease classifications through ongoing phenotypic, biomarker, and genetic research. Our study represents the largest national propensity‐matched analysis directly comparing these two disorders to date, offering population‐level insights that complement and extend findings from prior single‐center and consortium‐based investigations. Future prospective, multidimensional investigations integrating assessments of visceral hypersensitivity, gastric motor function, autonomic regulation, and psychosocial factors are needed to better delineate shared and disease‐specific mechanisms. Such integrative approaches may ultimately enable individualized, mechanism‐based therapies that improve patient outcomes and reduce health care burden.

This study has limitations inherent to retrospective analyses using administrative data for diagnostic and outcome coding. Although we applied rigorous inclusion criteria to minimize misclassification of gastroparesis and functional dyspepsia, coding inaccuracies may still occur. ICD‐10 codes for symptom encounters are not ideal for assessing symptom prevalence or severity. Furthermore, the database does not provide gastric emptying study results, precluding direct confirmation of delayed gastric emptying. Additionally, the database does not allow confirmation of whether clinical attributes or medication use are directly attributable to the condition of interest. Despite extensive propensity score matching, residual confounding from unmeasured variables may persist. Lastly, because patients with overlapping diagnoses of gastroparesis and functional dyspepsia were excluded to preserve cohort distinction, individuals who fall within the intermediate portion of the proposed gastroparesis–functional dyspepsia spectrum may not have been captured, which should be considered when interpreting our findings.

In conclusion, while gastroparesis and functional dyspepsia share overlapping clinical features, they exhibit distinct symptom distributions and therapeutic patterns at the population level. Our study cannot comment on whether these disorders represent separate entities or a continuum of the same underlying gastric neuromuscular dysfunction. However, the observed differences in symptom clusters and pharmacologic utilization suggest that distinguishing between the two in clinical practice remains valuable. Recognizing and addressing these differences through mechanism‐based and precision‐guided management will be essential for improving patient outcomes and advancing our understanding of these complex disorders.

## Author Contributions

**Sameer Rao**. – conceptualization, study design, interpretation of results, and co‐lead in original draft writing. **Vraj Shah**. – conceptualization, interpretation of results, and co‐lead in original draft writing. **Rohan Karkra**. – conceptualization, interpretation of results, and original draft writing. **Manas Gunani**. – data extraction and analysis, and manuscript review and editing. **Ritik Mahaveer Goyal**. – conceptualization, data interpretation, and manuscript review and editing. **Ahmed Al‐Khazraji**. – supervision, data interpretation, and manuscript review and editing. **Kaveh Hajifathalian**. – supervision, data interpretation, and manuscript review and editing. **Amanda A. Rupert**. – supervision, conceptualization, study design, project oversight, and lead in manuscript review and editing.

## Funding

The authors have nothing to report.

## Disclosure

Abstracts based on this study were presented at the American College of Gastroenterology Annual Scientific Meeting on 28th October 2025 in Phoenix, Arizona. No other disclosures to report.

Guarantor of the article**—**Sameer Rao.

## Ethics Statement

The authors have nothing to report.

## Conflicts of Interest

The authors declare no conflicts of interest.

## Supporting information


**Supplementary Table 1** Attributes and corresponding ICD‐10 (international classification of diseases, 10th revision), CPT (current procedural terminology), LOINC (logical observation identifiers names and codes), or Rxnorm codes used in the study.

## Data Availability

The data that support the findings of this study are available from the corresponding author upon reasonable request.
