# Supplementary material for: Exploring Clinical Similarities and Distinctions Between Gastroparesis and Functional Dyspepsia: A Propensity‐Matched Cohort Study
Source: Neurogastroenterol Motil. 2026 Jan 23;38(1):e70251. doi: 10.1111/nmo.70251 (PMC12831039; doi:10.1111/nmo.70251)
Supplement: Supplementary file 1 — Supplementary Table 1 Attributes and corresponding ICD‐10 (international classification of diseases, 10th revision), CPT (current procedural terminology), LOINC (logical observation identifiers names and codes), or Rxnorm codes used in the study. [file NMO-38-e70251-s001.docx]

**Supplementary Table** - Attributes and corresponding ICD-10 (international classification of diseases, 10th revision), CPT (current procedural terminology), LOINC (logical observation identifiers names and codes), or Rxnorm codes used in the study

| Attribute | ICD-10/CPT/LOINC/RxNorm |
| --- | --- |
|  |  |
| Abdominal distension | R14.0 |
| Abnormal weight loss | R63.4 |
| Acute myocardial infarction | I21 |
| Adjustment disorder | F43.2 |
| Alcohol use disorder | F10 |
| Angina pectoris | I20 |
| Anorexia | R63.0 |
| Anxiety | F41 |
| Aprepitant | 358255 |
| Aspiration pneumonitis | J69 |
| Asthma | J45 |
| Bipolar disorder | F31 |
| Cannabis use disorder | F12 |
| Celiac disease | K90 |
| Cerebral infarction | I63 |
| Chronic idiopathic constipation | K59.04 |
| Chronic ischemic heart disease | I25 |
| Chronic kidney disease | N18 |
| Chronic obstructive pulmonary disease | J44.9 |
| Commutated tomography, abdomen | 1010526 |
| Congenital absence, atresia and stenosis of large intestine | Q42 |
| Congenital absence, atresia and stenosis of small intestine | Q41 |
| Congenital hypertrophic pyloric stenosis | Q40.0 |
| Congenital malformation of stomach | Q40.3 |
| Crohn’s disease | K50 |
| Dependence on renal dialysis | Z99.2 |
| Dependence on respirator | Z99.1 |
| Depression | F32, F33 |
| Diphenhydramine | 3498 |
| Dronabinol | 10402 |
| Duodenal ulcer | K26 |
| Early satiety | E68.81 |
| Emergency department visit | 1013711 |
| Epigastric pain | R10.13 |
| Erythromycin | 4053 |
| Essential hypertension | I10 |
| Fatty liver | K76.0 |
| Fibrosis and cirrhosis of liver | K74 |
| Functional diarrhea | K59.1 |
| Functional dyspepsia | K30 |
| Gabapentin | 25480 |
| Gastrectomy | 43620, 43621, 43622, 43631 |
| Gastric emptying study | 78264, 78265, 78266 |
| Gastric ulcer | K25 |
| Gastritis and duodenitis | K29 |
| Gastroesophageal reflux disease | K21.9 |
| Gastrojejunostomy | 43820, 43825, |
| Gastroparesis | K31.84 |
| Gastrostomy | 43830, 43831 |
| Heart failure | I50 |
| Histamine antagonists | GA301 |
| Hospitalization | 1013659 |
| Hourglass stricture and stenosis of stomach | K31.2 |
| Hypothyroidism | E03.9 |
| Intestinal adhesions | K56.5 |
| Intussusception | K56.1 |
| Irritable bowel syndrome | K58 |
| Lipid metabolism disorder | E78 |
| Malignant neoplasm of stomach | C16 |
| Malnutrition | E40-46 |
| Metoclopramide | 6915 |
| Nausea/vomiting | R11, R11.0, R11.1, R11.2, R11.10, R11.11 |
| Nicotine dependence | F17 |
| Obstruction of duodenum | K31.5 |
| Ondansetron | 26225 |
| Opioid use disorder | F11 |
| Overweight and obesity | E66 |
| Post-traumatic stress disorder | F43.1 |
| Postprocedural intestinal obstruction | K91.3 |
| Prochlorperazine | 8704 |
| Promethazine | 8745 |
| Proton pump inhibitors (omeprazole, pantoprazole, esomeprazole, lansoprazole) | 7646, 40790, 283742, 17128 |
| Prucalopride | 2107310 |
| Pyloromyotomy | 43520 |
| Schizophrenia | F20 |
| Selective serotonin reuptake inhibitors (citalopram, escitalopram, duloxetine, paroxetine, sertraline) | 36437, 321988, 72625, 2556, 32937 |
| Serotonin-norepinephrine reuptake inhibitors (venlafaxine, desvenlafaxine) | 39786, 734064 |
| Severe malnutrition | E43 |
| Slow transit constipation | K59.01 |
| Tricyclic antidepressants | CN601 |
| Type 1 diabetes mellitus | E10 |
| Type 2 diabetes mellitus | E11 |
| Ulcerative colitis | K51 |
| Upper endoscopy | 43235, 43239 |
| Volvulus | K56.2 |
|  |  |
